# Developmental Trends in Serum Iron, Transferrin, and Transferrin Saturation From Birth to 12 Months

**DOI:** 10.1111/apa.70558

**Published:** 2026-04-26

**Authors:** Sara Marie Larsson, Lena Hellström‐Westas, Ulrica Askelöf, Cecilia Götherström, Magnus Domellöf, Ola Andersson

**Affiliations:** ^1^ Department of Clinical Chemistry Halland Hospitals Varberg Sweden; ^2^ Department of Clinical Sciences, Lund Paediatrics, Lund University Lund Sweden; ^3^ Department of Women's and Children's Health Uppsala University Uppsala Sweden; ^4^ Department of Clinical Science, Intervention and Technology, Division of Obstetrics and Gynaecology Karolinska Institutet Stockholm Sweden; ^5^ Department of Gynaecology and Reproductive Medicine Karolinska University Hospital Stockholm Sweden; ^6^ Department of Clinical Sciences Paediatrics, Umeå University Umeå Sweden

**Keywords:** circadian rhythm, delayed cord clamping, diurnal variation, hypoferremia, paediatric reference intervals

## Abstract

**Aim:**

There is a need for updated reference intervals for iron status biomarkers during infancy. This study aimed to investigate reference interval trends and diurnal variation of iron, transferrin, and transferrin saturation in infants subjected to delayed cord clamping at birth (DCC).

**Methods:**

Data analysis from population‐based Swedish studies, including 362 term‐born infants subjected to DCC ≥ 60 s. The 2.5th and 97.5th percentiles were calculated from serum samples: In cord blood, at 48–118 h, four months and 12 months. We used Spearman's rho to test for associations and Student's *t*‐test to compare groups.

**Results:**

Iron concentrations in cord blood were initially high, 14–41 μmol/L, and had decreased by 48–118 h to 6–16 μmol/L, remaining mainly constant thereafter. Conversely, early transferrin concentrations were low. Transferrin slowly increased; concentrations at 4 months were positively associated with the average weight gained per day, rho = 0.46, *p* < 0.001. A small diurnal difference in iron was observed at 48–118 h: Samples collected between 10:30–11:59 were, on average, 1.4 μmol/L (95% CI −2.8 to −0.0) lower compared with samples collected between 15:30–19:59.

**Conclusion:**

Developmental trends in iron, transferrin, and transferrin saturation in the first year of life need to be considered in the interpretation of test results from infants.

## Key Points

- Modern, well‐defined normative data describing iron, transferrin, and transferrin saturation during infancy are lacking.
- Our study reports reference intervals and diurnal trends in infants who had undergone delayed clamping of the cord at birth.
- Developmental trends rather than discrete reference intervals need to be considered.

**Abbreviations**
CI: confidence interval
DCC: delayed cord clamping
TSat: transferrin saturation

## 1 Introduction

It is challenging to determine iron status in infants [1]. The most widely used approach is to measure ferritin concentrations in plasma. Ferritin reflects the amount of iron stores in the absence of infection or inflammation. However, it does not reflect the availability of biologically accessible iron in the circulation, which is reduced in states of functional iron deficiency. Also, ferritin results can be difficult to interpret in the context of iron overload. For these aspects, the additional measurement of iron, transferrin, and transferrin saturation (TSat) can sometimes be required.

Iron measured in plasma has long been considered to exhibit diurnal variation and has subsequently been considered to be an unreliable biomarker of systemic iron status. In adults, iron concentrations peak before noon and decrease in the afternoon [2] where the pattern seems to follow the erythropoietic activity in an opposing phase [3]. Iron concentrations reach a nadir when erythropoiesis is at its peak [3]. As for infants, present knowledge is insufficient to determine the stage at which diurnal variation develops. Infant sleep patterns are often irregular, and the contributions from daylight or diet may be influential.

In addition, assessments of iron, transferrin, and TSat during infancy may be particularly difficult because of the paucity of well‐defined normative data. Although there are several studies published in the past three decades [4, 5, 6, 7], these studies lack detailed information about infant population characteristics. Maternal health, pregnancy complications, mode of delivery, gestational age at birth, and infant growth [8] are variables which could all play a significant role. In addition, modern reference interval studies have so far not taken the timing of umbilical cord clamping at birth into account. The latter variable has been shown to be beneficial and significantly increase iron stores during an infant's first year [9].

The current knowledge about infant reference intervals, therefore, primarily relies on investigations conducted in the 1950s and 1970s [10, 11, 12, 13]. In these early studies, TSat was defined by using assays measuring total iron‐binding capacity. This means that the measurements could have been prone to drawbacks [14]. The assays had issues with harmonisation, which contributed to a large between‐assay variation. Also, analysis procedures involving adding excess amounts of iron salts to the sample can cause nonspecific binding of iron to other plasma proteins, making the results difficult to interpret [14]. In modern laboratories, these methodological challenges have been overcome by transitioning to use assays measuring the transferrin concentration. These assays have been successfully standardised by both the establishment of a certified international reference material [15] and an international reference method [16].

The lower infant reference limit of TSat, obtained in 1977 using the older methodology, was only 10%. In infants with sufficient iron stores and no infection or inflammation, this finding was in sharp contrast to the knowledge of compromised adult erythropoiesis already at TSat lower than 15%–20% [17]. The low limit was unexpected as the infants were fed a high‐dose iron formula, and the limit remained low also when using exclusion criteria based on other iron status biomarkers [10]. Therefore, considering the methodological development since the 1970s and these unexpected historical findings, there is a need for reinvestigation of reference limits.

This study aimed to increase knowledge about iron, transferrin, and TSat during infancy in a large cohort of infants subjected to delayed clamping of the cord at birth. We investigated potential diurnal differences, the influence of growth on transferrin concentrations, and used the currently preferred analytical methodology to describe reference intervals during the first year of life.

## 2 Methods

### 2.1 Study Population

This study comprised infants born at two Swedish hospitals: The County Hospital of Halland in southern Sweden and the Karolinska University Hospital Huddinge in Stockholm. The data were collected from 2008 to 2013, as part of three clinical studies that assessed the effects of the timing of cord clamping [18, 19, 20].

The infants were born at 37 + 0 to 41 + 6 weeks of gestation, and the mothers had to be healthy, non‐smokers, with no haemolytic disease and not receiving antidepressants, anticonvulsants, cortisone, thyroid hormone, insulin or chemotherapy. They were only included if they had a normal singleton term pregnancy, with no diabetes, pre‐eclampsia, prolonged rupture of membranes or signs of infection. Perinatal circumstances were uneventful.

At the County Hospital of Halland, blood samples were collected at four time points: At birth (cord blood), in conjunction with metabolic screening at 48–118 h, at four months and at 12 months. At the Karolinska University Hospital, blood samples were collected at two time points: At birth (cord blood) and at four months.

Data were included in the reference interval calculations if the infants had undergone delayed cord clamping at 60 s or more. Also, a cutoff of 2.5 mg/L was applied for a concurrently measured C‐reactive protein to exclude interference from the acute‐phase response. Details describing the study population are presented in Table 1.

**TABLE 1:** Characteristics of the study population.

Time points	Clinical information	Reference interval population Inclusion and exclusion details
At birth: Umbilical cord blood		Umbilical cord clamping ≥ 60 s Infants: *n* = 362 *Exclusions:* *CRP* > *2.5 mg/L n = 5* *CRP result missing n = 6*
		Eligible results: Iron *n* = 351 Transferrin *n* = 351
1–6 h	Assessed by the midwife: 1 h and 6 h after birth. Record of feeding and respiratory symptoms.	
72 h	Examination by a physician in accordance with clinical routines.	
Halland hospital cohort only 48–118 h	Blood sampling in conjunction with metabolic screening.	Umbilical cord clamping ≥ 60 s Infants: *n* = 175 *Exclusions:* *Sampling or instrumental issues (*e.g., *hemolysis or clot):* *Both iron and transferrin n = 31* *Iron only n = 12* *Transferrin only n = 2*
		Eligible results: Iron *n* = 132 Transferrin *n* = 142
Follow‐up visit 4 months	Blood sampling, weight and length measurement.	Umbilical cord clamping ≥ 60 s Infants returning for assessment: *n* = 319 *Exclusions:* *CRP* > *2.5 mg/L n = 20* *CRP result missing n = 1* *Sampling or instrumental issues (*e.g., *hemolysis or clot) n = 14*
		Eligible results: Iron *n* = 284 Transferrin *n* = 284
Halland hospital cohort only Follow‐up visit 12 months	Blood sampling, weight and length measurement	Umbilical cord clamping ≥ 60 s Infants *n* = 157 *Exclusions:* *CRP* > *2.5 mg/L n = 35* *CRP result missing n = 2*
		Eligible results: Iron *n* = 120 Transferrin *n* = 120

### 2.2 Specimen Collection and Handling

Blood was collected in serum separator tubes. Halland used the BD Vacutainer (Becton Dickinson and Company, Devon, UK). Karolinska used S‐Monovette tubes (Sarstedt AG & Co., Oberbergischer Kreis, Germany). At Karolinska University Hospital, the serum was kept at −70°C before being sent for analysis.

At four and 12 months, EMLA, a local eutectic mixture of local anaesthetics containing lidocaine 2.5% and prilocaine 2.5% (AstraZeneca, Södermanland, Sweden) was applied before blood sampling. The time of day when blood was collected (hour: minute) was registered for the Halland cohort.

### 2.3 Laboratory Analysis

The serum samples were analysed for iron, transferrin, ferritin and C‐reactive protein on the Cobas 6000 instrument platform (Roche Diagnostics, Basel, Switzerland) at the clinical chemistry laboratory at the County Hospital of Halmstad, Sweden. TSat was calculated using the formula: Iron/transferrin x 25.157. The iron assay was traceable to the international standard reference material SRM937, while the transferrin assay was traceable to Bureau Communautaire de Référence BCR470/CRM 470. The laboratory was accredited according to SE‐EN ISO/IEC 17025:2005 and participated in inter‐laboratory external proficiency testing schemes.

### 2.4 Calculations and Statistical Analysis

Reference intervals with 90% confidence intervals were calculated non‐parametrically as 2.5th and 97.5th percentiles, according to ***EP28*** ‐A3c Clinical and Laboratory Standards Institute guidelines [21]. The widths of the reference intervals were defined as the upper reference limit minus the lower limit. The lack of overlap of confidence intervals was considered a statistically significant difference. The association between transferrin concentrations and the average weight gain per day at four and 12 months, respectively, was assessed using Spearman's rank correlation.

To estimate the potential role of diurnal variation, groups were retrospectively defined based on the time of day when samples were obtained (Table *2*). Samples at 48–118 h were obtained continuously around the clock in conjunction with metabolic screening, while samples at four and 12 months were obtained during regular office hours. The defined time frames for investigation of diurnal variation were selected based on a study of adults, which presents continuously increasing iron and TSat concentrations from early morning with a peak at the hour before noon (2). Thereby, groups at 48–118 h, four months and 12 months were defined from samples obtained at 10:30 to 11:59. The study in adults also showed a continuously decreasing trend in the afternoon, reaching its lowest concentration first around 20:00 (2). The 48–118 h samples were collected over 24 h, and a comparative group was defined based on samples obtained from 15:30 to 19:59. At four and 12 months, samples were collected during office hours, why the second group was investigated using a somewhat shorter time frame of 15:30 to 17:50. The groups with the time frames corresponding to peak and lower concentrations were compared using the Student's independent samples *t*‐test, with a significance level of 5%.

**TABLE 2:** Comparison of the mean concentration of iron (Fe), transferrin (Tfr), and transferrin saturation (TSat) based on groups defined by time of day when the blood sample was obtained.

Sampling time point (*n*)	Time of day	Mean (SD)		
		Iron (μmol/L)	Transferrin (g/L)	TSat (%)
**48–118 h** (*n* = 132)	03:00–22:49	9.9 (2.7)	1.75 (0.20)	22.9 (6.7)
*A1. Fe (n = 38) Tfr (n = 41)*	10:30–11:59	10.6 (2.6)	1.77 (0.25)	24.6 (7.4)
*A2. Fe (n = 35) Tfr (n = 43)*	12:00–15:29	10.3 (2.8)	1.75 (0.17)	23.4 (6.5)
*A3. Fe (n = 19) Tfr (n = 20)*	15:30–19:59	9.2 (2.2)	1.72 (0.20)	21.5 (6.1)
*A4. Fe (n = 30) Tfr (n = 30)*	20:00–10:29	9.5 (2.7)	1.72 (0.18)	22.1 (6.3)
*A1* vs. *A3* *Mean difference (95% CI)* *p‐value*		−1.4 (−2.8 to –0.0) *p* = 0.05	−0.05 (−0.18–0.07) *p* = 0.39	−3.1 (−7.0–0.9) *p* = 0.12
**4 months** (*n* = 151)	10:34–17:49	10.4 (2.9)	2.28 (0.31)	18.7 (6.2)
*B1. (n = 30)*	10:34–11:59	11.8 (3.3)	2.24 (0.31)	21.7 (7.8)
*B2. (n = 64)*	12:24–15:29	10.4 (3.2)	2.28 (0.32)	18.7 (6.3)
*B3. (n = 53)*	15:30–17:49	9.6 (2.0)	2.28 (0.29)	17.2 (4.4)
*B1* vs. *B3* *Mean difference (95% CI)* *p‐value*		−2.1 (−3.3 to −1.0) *p* = 0.0004	0.04 (−0.10–0.18) *p* = 0.55	−4.4 (−7.1 to −1.8) *p* = 0.001
**12 months** (*n* = 118)	10:38–17:02	10.3 (3.3)	2.74 (0.28)	16.2 (5.8)
*C1. (n = 37)*	10:38–11:59	12.1 (4.1)	2.73 (0.30)	18.0 (6.0)
*C2. (n = 44)*	12:30–15:29	11.3 (2.9)	2.68 (0.27)	17.1 (5.4)
*C3. (n = 37)*	15:30–17:02	9.3 (3.3)	2.82 (0.27)	13.4 (5.0)
*C1* vs. *C3* *Mean difference (95% CI)* *p‐value*		−2.8 (−4.5 to −1.1) *p* = 0.002	0.10 (−0.04–0.23) *p* = 0.16	−4.6 (−7.2 to −2.0) *p* = 0.0006

*Note:* Missing data specification with exact blood sampling time (hour: minute) excl. from calculations: At 48–118 h, *n* = 10, 4 months *n* = 4, 12 months *n* = 2.

The reference interval calculations were carried out using Analyse‐it for Microsoft Excel 5.90 (Analyse‐it Software Ltd, West Yorkshire, UK), and the statistical analyses were carried out using SPSS 31.0 (SPSS, New York, USA).

### 2.5 Ethics

Ethical approval was provided by the regional research ethics committee at Lund University (41/2008, 344/2009) and by the regional ethical review board in Stockholm (2011/2142‐31/3). Written parental/guardian consents were obtained.

## 3 Results

The study population was based on 362 infants (53% girls) born at a mean gestational age of 40.0 ± 1.1 weeks and a mean birth weight of 3620 ± 459 g. All infants were within the normal weight‐for‐age growth standards [22]. The mean weight at four months was 6 978 ± 880 g, and this was measured at a median age of 122 days (interquartile range 120–127 days, minimum 109 to max 154 days). The mean weight at 12 months was 10 063 ± 1 040 g, measured at a median age of 364 days (interquartile range 361 to 368 days, minimum 350 to maximum 396 days).

### 3.1 Reference Intervals

**FIGURE 1:**
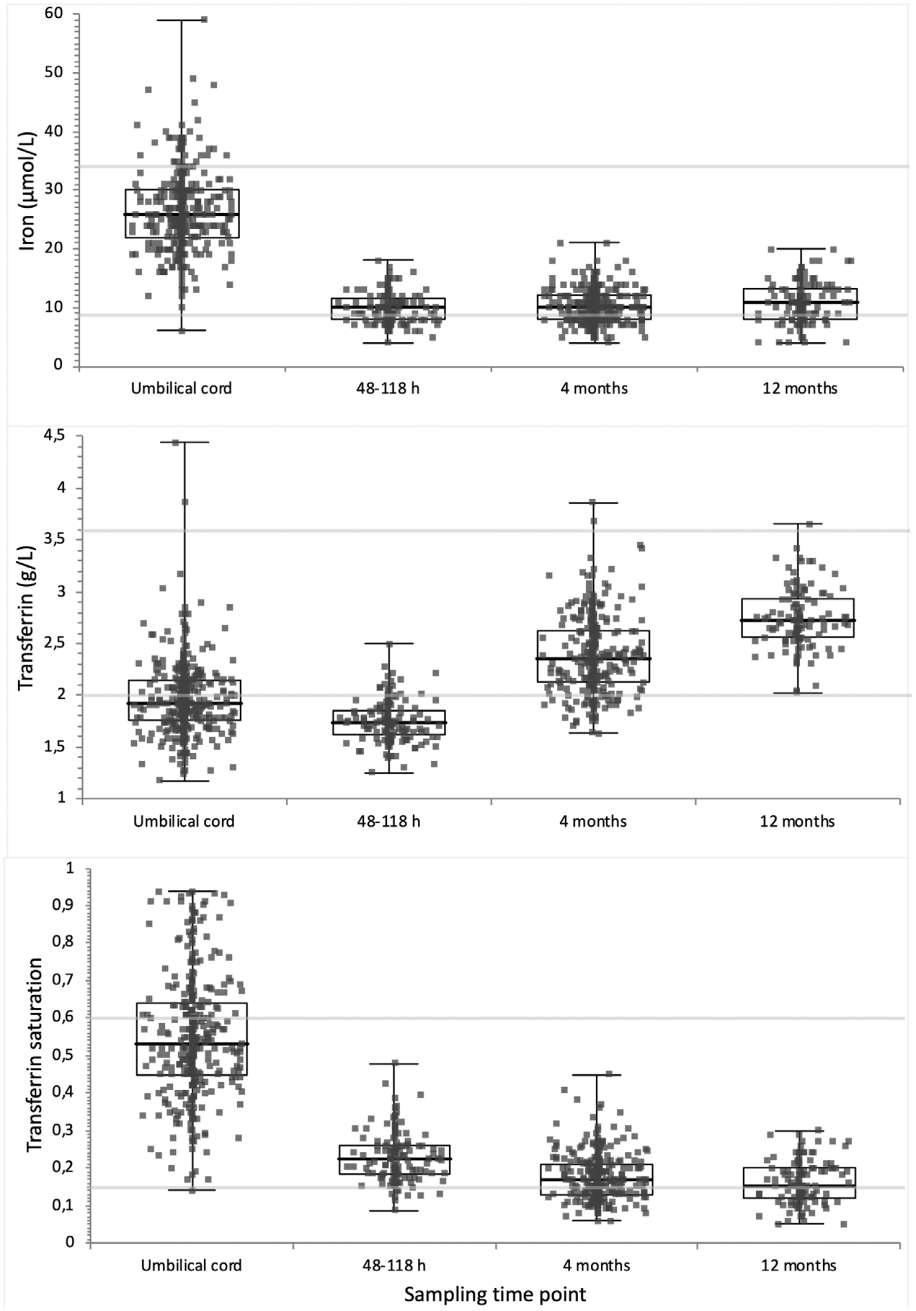
Iron, transferrin and TSat in 362 full‐term infants presented as combined dot and skeletal boxplots showing the minimum, first quartile, median, third quartile and maximum. Adult reference intervals from the Nordic reference interval study [23] as comparison (grey).

Reference intervals for iron, transferrin and TSat in samples obtained from the cord, at 48–118 h, at four months and at 12 months are presented in Table 3. The data are visually presented as bee‐swarm scatter plots in Figure 1.

**TABLE 3:** Reference intervals for iron, transferrin and transferrin saturation in infants at four time points in the first year of life.

Sampling time point	Median (IQR)	Lower (2.5th percentile)	Lower 90% CI	Upper (97.5th percentile)	Upper 90% CI
**Iron (μmol/L)**					
A. Umbilical cord	26 (22–30)	**14**	(12–16)	**41**	(39–47)
B. 48–118 h	10 (8–12)	**6**	(4–6)	**16**	(15–18)
C. 4 months	10 (8–12)	**5**	(5‐6)	**17**	(16–21)
D. 12 months	11 (8–13)	**4**	(4‐5)	**18**	(17–20)
**Transferrin (g/L)**					
A. Umbilical cord	1.92 (1.76–2.15)	**1.35**	(1.28–1.44)	**2.79**	(2.64–3.03)
B. 48–118 h	1.73 (1.62–1.85)	**1.38**	(1.26–1.43)	**2.20**	(2.10–2.50)
C. 4 months	2.36 (2.12–2.62)	**1.76**	(1.70–1.83)	**3.21**	(3.07–3.44)
D. 12 months	2.72 (2.56–2.93)	**2.10**	(2.03–2.37)	**3.33**	(3.29–3.65)
**Transferrin saturation (%)**					
A. Umbilical cord	53 (45–64)	**24**	(18–28)	**91**	(88–93)
B. 48–118 h	23 (18–26)	**12**	(9–13)	**39**	(35–48)
C. 4 months	17 (13–21)	**8**	(7–9)	**35**	(30–41)
D. 12 months	15 (12–20)	**6**	(5–7)	**29**	(27–30)

*Note:* data are presented as 2.5th and 97.5th percentiles with 90% confidence intervals (CI).

### 3.2 Iron

The iron reference limits in cord blood were 2–4 times higher than the three later time points. There was a large variation between individuals in the cord blood, contributing to a large width of the reference interval (26 μmol/L) compared to the later time points (10–14 μmol/L).

### 3.3 Transferrin

In contrast to iron, transferrin concentrations were low at birth and increased with postnatal age. The widths of the reference intervals were the same in cord blood and at four months (approximately 1.5 g/L), but lower at 48–118 h (0.8 g/L).

**FIGURE 2:**
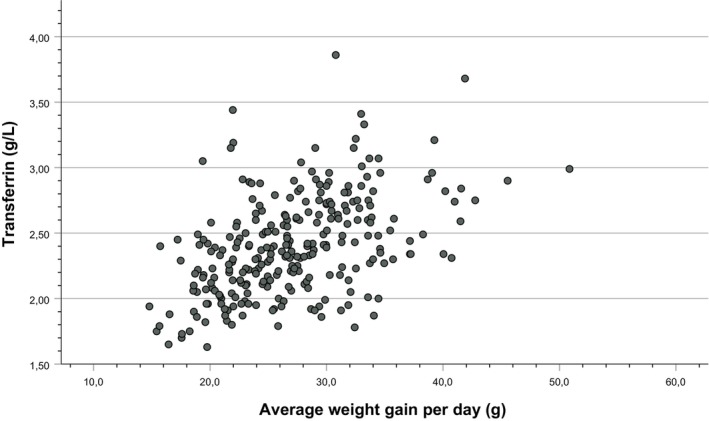
The association between the average weight gain per day (g) in the first 4 months and the transferrin concentration in serum at the age of 4 months.

The transferrin concentration at 4 months was significantly correlated with average weight gain per day, rho = 0.48, *p* < 0.001 (Figure 2) and to a lesser extent with infant postnatal age, rho = 0.13, *p* < 0.03. Mean weight gained from birth to 4 months was 3 345 g (SD 753). At 12 months, no significant association was observed between the transferrin concentration and the average weight gain per day, rho = 0.15, *p* = 0.11.

### 3.4 Transferrin Saturation

The TSat in cord blood ranged from 24% to 91% (2.5th and 97.5th percentiles). By 48–118 h, the levels at both the upper and the lower reference limits had rapidly decreased to approximately half (12%–39%). Further decreases were observed at both the upper and the lower reference limits at four and 12 months (Table 3). At 12 months, the very low reference limit (5%) remained unaffected when we restricted the reference interval population by only including 41 infants with a concurrently measured ferritin of ≥ 40 μg/L. The widths of the reference intervals were relatively consistent between time points: 27% at 48–118 h, 27% at four months and 23% at 12 months.

### 3.5 Diurnal Variation

The comparison across the groups with before‐noon samples and late‐afternoon samples showed a statistically significant overall difference in iron concentrations at all three investigated time points (Table 2). At 48–118 h, iron concentrations were on average 1.4 μmol/L lower in the group with blood sampling before noon (95% CI −2.8 to −0.0) compared with the group with blood samples in the late afternoon. At four months, the difference was −2.1 μmol/L (95% CI −3.3 to −1.0) and at 12 months −2.8 μmol/L (95% CI −4.5 to −1.1). For transferrin, no differences were observed. As for TSat, there was an average diurnal difference of −3.1% at 48–118 h, which was approaching statistical significance (95% CI −7.0 to 0.9). The differences were statistically significant at both four and 12 months: −4.4% (95% CI −7.1 to −1.8) and −4.6% (95% CI −7.2 to −2.0), respectively.

## 4 Discussion

This study reports dynamic changes in the regulation of transferrin and transferrin saturation in infants during their first year of life. The transferrin saturation decreased with increasing age, following increased serum concentrations of the transport protein transferrin after birth. The reference limits for iron remained mainly constant.

Our study also suggests that the diurnal variation of serum iron is already established a few days after birth. The average differences between samples collected before noon and samples collected later in the afternoon were, however, small. Our observation could nevertheless indicate that a diurnal cycle of iron homeostasis is tightly regulated already in early life.

The transferrin concentrations at four months were positively correlated with the average weight gain per day. This indicates that the concentration during this time frame can be associated with the growth rate. Like several other plasma proteins, transferrin has been reported to increase progressively with both gestational and postnatal age [24].

A low transferrin concentration has been associated with a prioritisation towards tissue iron rather than erythropoietic requirements, such as in congenital atransferrinaemia. This observation could be of interest with regard to future studies on early infant iron depletion, on prioritisation towards erythropoiesis and on the neurodevelopmental aspects of infant iron deficiency. To the best of our knowledge, further detailed research is needed on the regulatory mechanisms of transferrin concentrations during the prenatal period and early life, with regard to age, growth and nutrition.

Our findings on TSat excluded methodological reasons as the underlying cause for the historically low reference limits during the first year of life. The low limit found in our large cohort of infants at birth, after delayed cord clamping, is intriguing. Erythropoiesis in adults is iron‐restricted if TSat decreases below 15%–20% [17]. The observed difference between our study and adults calls for future studies to clarify the potential iron‐regulating biochemical pathways involved and the risk for more long‐term sequelae, such as anaemia.

In contrast, exceedingly high TSat was present in cord blood. The upper TSat reference limit of 91% in cord blood is far above levels that would indicate a severe iron overload in adults. Levels of potentially oxidatively reactive non‐transferrin‐bound iron have been reported to increase sharply in a hyperbolic pattern at 70% [25] and in the context of hereditary hemochromatosis, already a TSat of 45% is regarded as elevated [25, 26]. More research is needed into the possible roles that a high saturation of transferrin can play in prenatal development.

In addition, when we compared cord blood TSat between infants, we found major differences in levels. The cord blood values ranged from 24% for the 2.5th percentile to 91% for the 97.5th. To the best of our knowledge, the prenatal and perinatal factors involved have not been explored, and more research is needed to investigate the potential roles of factors such as maternal iron status.

We also found a rapid 2–4‐fold decrease in iron concentrations 48–118 h after birth. Studies have suggested that a postnatal physiological hypoferremia forms part of the innate immune system, as the growth rate of several common bacteria has been shown to increase with increasing TSat values [27].

### 4.1 Strengths and Limitations

This study has a number of strengths. We used modern assays and adhered to the Clinical and Laboratory Standards Institute guidelines to calculate reference intervals. Parents had stated that their children were well, and we excluded results with an increased concentration of C‐reactive protein. Also, the infants had undergone delayed cord clamping at birth, which has confirmed benefits. In addition, well‐defined postnatal time‐points were used.

The study was, however, limited by design to accurately investigate diurnal variation. Optimally, repeated blood sampling in the same individual over 24 h is required, a design which, in infants, is ethically unjustifiable.

## 5 Conclusion

This study presents reference intervals for iron, transferrin, and transferrin saturation in a large cohort of infants who had undergone delayed cord clamping at birth. The transferrin saturation decreased with increasing age. Rapid weight gain during the first four months was associated with higher transferrin concentrations at this time point. Our study also indicates that diurnal variation in iron and transferrin saturation has developed already 48–118 h after birth. In conclusion, in the interpretation of test results from infants, developmental changes in transferrin and transferrin saturation in the first year of life need to be considered.

## Author Contributions

**Sara Marie Larsson:** conceptualization, investigation, funding acquisition, writing – original draft, writing – review and editing, formal analysis. **Magnus Domellöf:** writing – review and editing, resources, investigation, conceptualization. **Ola Andersson:** writing – review and editing, resources, data curation, investigation, conceptualization. **Lena Hellström‐Westas:** writing – review and editing, resources, investigation, conceptualization. **Ulrica Askelöf:** data curation, writing – review and editing, resources, investigation. **Cecilia Götherström:** writing – review and editing, data curation, resources, investigation, conceptualization.

## Funding

This work was supported by Region Halland (HALLAND‐1011927).

## Conflicts of Interest

The authors declare no conflicts of interest.

## Data Availability

The data that support the findings of this study are available on request from the corresponding author. The data are not publicly available due to privacy or ethical restrictions.
